# Unraveling Nanomaterials in Biomimetic Mineralization of Dental Hard Tissue: Focusing on Advantages, Mechanisms, and Prospects

**DOI:** 10.1002/advs.202405763

**Published:** 2024-08-29

**Authors:** Danni Dai, Dan Li, Chao Zhang

**Affiliations:** ^1^ Stomatological Hospital School of Stomatology Southern Medical University Guangzhou 510280 China

**Keywords:** biomimetic mineralization, nanomaterials, remineralization

## Abstract

The demineralization of dental hard tissue imposes considerable health and economic burdens worldwide, but an optimal method that can repair both the chemical composition and complex structures has not been developed. The continuous development of nanotechnology has created new opportunities for the regeneration and repair of dental hard tissue. Increasingly studies have reported that nanomaterials (NMs) can induce and regulate the biomimetic mineralization of dental hard tissue, but few studies have examined how they are involved in the different stages, let alone the relevant mechanisms of action. Besides their nanoscale dimensions and excellent designability, NMs play a corresponding role in the function of the raw materials for mineralization, mineralized microenvironment, mineralization guidance, and the function of mineralized products. This review comprehensively summarizes the advantages of NMs and examines the specific mineralization mechanisms. Design strategies to promote regeneration and repair are summarized according to the application purpose of NMs in the oral cavity, and limitations and development directions in dental hard tissue remineralization are proposed. This review can provide a theoretical basis to understand the interaction between NMs and the remineralization of dental hard tissue, thereby optimizing design strategy, rational development, and clinical application of NMs in the field of remineralization.

## Graphical abstract

Due to their nanoscale dimensions and excellent designability, nanomaterials can regulate the different stages of biomimetic mineralization of dental hard tissue. They play a corresponding role in the function of the raw materials for mineralization, mineralized microenvironment, mineralization guidance, and the function of mineralized products, facilitating the repair of both the chemical composition and complex structures of dental hard tissue.

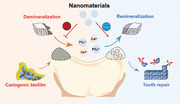

## 1 Introduction

**Figure 1:**
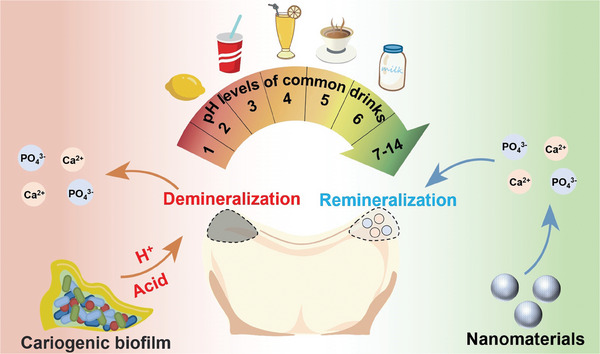
Demineralization and remineralization of dental hard tissue as well as the pH of daily beverages. Acid production by cariogenic bacteria and an acidic diet lead to the dissolution of calcium and phosphorus ions and the demineralization of dental hard tissues. The introduction of NMs improves the local acidic microenvironment, leading to the redeposition of calcium and phosphorus ions and the remineralization of dental hard tissues.

Dental hard tissue is highly mineralized and composed of hydroxyapatite (HA) crystals, organic molecules (primarily collagenous and non‐collagenous proteins), and a small amount of water.^[^ ^1^, ^2^ ^]^ The degree of mineralization is closely related to chewing, digestion, and smile aesthetics. Demineralization of dental hard tissue (including enamel, dentin, and cementum) caused by cariogenic bacteria, acidic diet, and acid reflux is among the most prevalent diseases globally and imposes considerable health and economic burdens in both developed and developing countries.^[^ ^3^ ^]^ The demineralization of enamel may lead to the formation of white spot lesions or incipient caries that degrade dental aesthetics and function. Mild demineralization of dentin may l aead to hypersensitivity, while severe demineralization may result in deep caries and pulp involvement.^[^ ^1^ ^]^ In the elderly, gingival recession at the tooth neck and the exposure of roots, together with reduced salivation, lead to large and shallow caries on the thin cementum which are usually difficult to repair.^[^ ^4^ ^]^ The demineralization and remineralization of dental hard tissue are two antagonistic processes of dissolution and redeposition of Ca^2+^, OH^−^ and PO_4_^3−^, both of which occur concurrently or sequentially under physiologic conditions (**Figure** **1**).^[^ ^5^, ^6^ ^]^ However, when HA dissolves at pH < 5.5, the acellular hard tissue can scarcely redeposit equivalent amounts of HA and repair itself within a brief time span, because calcium and phosphorus ion concentrations of salivary are low. This imbalance leads to demineralization‐induced defects, such as white spot lesions and caries. Therefore, clinical intervention is urgently needed to reverse dental demineralization and promote remineralization.^[^ ^7^ ^]^

Pro‐mineralization drugs approved for clinical use primarily include fluoride, casein phosphopeptide amorphous calcium phosphate complex (CPP‐ACP), self‐assembling peptide P11‐4, nano‐HA, bioactive glass, etc.^[^ ^8^, ^9^ ^]^ Fluorapatite formed by the bonding of fluorine and HA can improve resistance to acid. However, because fluorine does not provide calcium and phosphorus for remineralization, it cannot restore the mechanical properties of demineralized tissue.^[^ ^10^ ^]^ In addition, long‐term application carries a risk of adverse reactions such as fluorosis.^[^ ^11^ ^]^ Although CPP‐ACP can provide raw materials for remineralization, its clinical efficacy is limited by instability and poor permeability.^[^ ^12^, ^13^ ^]^ More importantly, both fluorapatite and CPP‐ACP lack stage control of the remineralization process, and restore the structure and mechanical properties of complex dental hard tissue ineffectively.^[^ ^14^ ^]^ Self‐assembling peptide P11‐4 has a high affinity with calcium ions and could simulate the enamel matrix protein to guide biomimetic enamel remineralization. However, its work is highly dependent on saliva to provide effective ions, limiting its ability to treat xerostomia patients.^[^ ^15^ ^]^ Bioactive glass, mainly composed of calcium oxide, phosphorus oxide, silicon dioxide (SiO_2_), and so on, aids in remineralization after being dissolved to offer calcium and phosphorus ions. Regrettably, these ions without control easily rapidly aggregate on the surface of enamel and spontaneously convert into crystals, leading to a limited transformation rate for biomimetic mineralization. In the past decade, the development of nanotechnology and biomimetic mineralization have opened new opportunities for the regeneration and repair of dental hard tissue.^[^ ^16^ ^]^ nHA, one of the typical NMs in remineralization, has a strong surface adsorption force on hard tooth tissue with its high surface chemical activity and homogeneity with apatite crystal in the tooth.^[^ ^17^ ^]^ However, isolated nHA is limited to code with demineralization to a minor degree. Moreover, nHA is mainly used for enamel repair without substantial defect, while it has component boundaries with dentin and cementum which have more complex structures, thus not sufficiently qualified for their repair. This review, focusing on the theme of NMs of which nHA is an important member, summarized the current progress of the combination of nHA and other NMs attempting to broaden the mineralization efficiency and application range of nHA, which will be described in detail later. The mechanisms of nano‐HA and its derivatives/analogs, polyelectrolyte‐based nanomaterials (NMs) represented by poly(amidoamine) (PAMAM), and mesoporous NMs such as silica and calcium silicate in mineralization have been studied extensively.^[^ ^18^ ^]^ On one hand, as drug delivery systems, NMs contribute to the efficient delivery and controlled release of mineral ions to demineralized sites.^[^ ^19^, ^20^ ^]^ On the other hand, the excellent structural designability of NMs enables highly efficient mimicry of organic nucleation templates, promoting nucleation mineralization and inducing crystal assembly.^[^ ^21^, ^22^ ^]^ Last but not least, NMs show more effective antibacterial properties compared to antibiotics, which are imperative to prevent cariogenic bacteria from producing acid and promoting mineralization. Their nanoscale dimensions endow them with excellent photothermal, photocatalytic, and mechanical properties, thus indirectly improving remineralization efficiency by intelligently inhibiting bacteria.

Recent studies have reported the excellent activity of NMs in promoting the regeneration and repair of dental hard tissue.^[^ ^23^, ^24^ ^]^ Several reviews have also provided a combined list of NMs with outstanding effects; however, mechanisms of action have not been summarized.^[^ ^15^, ^25^, ^26^ ^]^ In particular, the ways by which NMs achieve biomimetic remineralization, the similarities and differences between the mechanisms of different NMs, and the features that have potential clinical applications have not been discussed systematically. This review summarizes the advantages of NMs in promoting the remineralization of different dental hard tissues through the physicochemical process and discusses the mechanism of NMs from the process of biomimetic mineralization. However, those NMs promoting the occurrence of remineralized tissues by acting on oral cells are not included in the scope of this review. Moreover, we summarize the design strategies of NMs to promote regeneration and repair according to their purpose and applications in the oral cavity and propose limitations and directions for the development of dental hard tissue remineralization. This review can provide a theoretical basis for a better understanding of the interaction between NMs and the remineralization of dental hard tissues, thereby optimizing design strategy, rational development, and clinical applications of NMs in the field of remineralization.

## 2 Advantages of NMs in Biomimetic Remineralization

**Figure 2:**
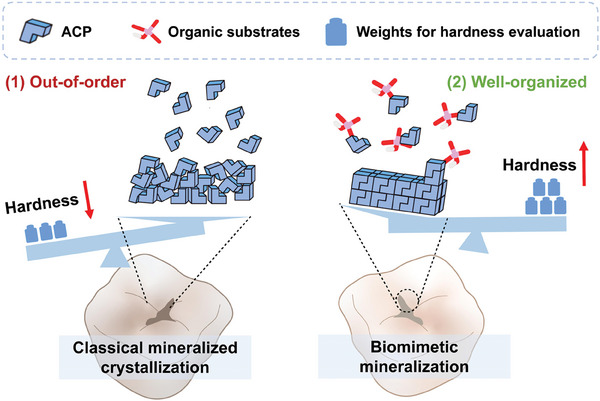
Comparison of the internal crystal phase structure, external topology, and weight‐bearing capacity of crystals in the theories of classical mineralized crystallization and biomimetic mineralization. ACP: amorphous calcium phosphate.

Dental hard tissue is a product of biomineralization. The classical mineralized crystallization theory holds that the initially formed crystal nuclei complete the replication and growth of the unit cell through interionic adsorption and eventually grow into a macroscopic crystal.^[^ ^27^ ^]^ The resultant remineralization scheme (based on various saturated calcium‐phosphorus mineralization solutions) is highly dependent on the existing apatite crystal nucleus and simply promotes calcium and phosphorus deposition, which is insufficiently targeted. Replication of the naturally ordered HA crystal by the internal crystal phase structure and external topology of the regenerated mineralized structure is difficult, and thus the resultant mechanical properties are inadequate. However, from the concept of biomimetic mineralization, the induction and regulation of mineralization entails the deposition of appropriate ions at specific sites under the control of organic substrates and the formation of a complex hierarchical structure after nucleation, growth, and assembly; wherein, amelogenin, collagenous fiber, and non‐collagen protein serve as templates for crystal nucleation (**Figure** **2**).^[^ ^27^, ^28^, ^29^ ^]^ NMs have an excellent size advantage in the biomimetic induction of dental hard tissue mineralization. Specifically, the sizes of the initially formed amorphous calcium phosphate (ACP) clusters (≈1 nm),^[^ ^30^ ^]^ matrix protein (≈10 nm),^[^ ^31^ ^]^ and interval of collagen fiber (≈40 nm)^[^ ^32^ ^]^ and amelogenin (≈10 nm)^[^ ^33^ ^]^ are all nanoscales. NMs are conducive to the complete restoration of remineralized tissue from chemical composition to complex structure.^[^ ^34^ ^]^ However, because the structures and functions of enamel, dentin, and cementum differ, the nucleation templates and organic substrates of biomineralization are dissimilar, and the roles of NMs also vary.

### 2.1 NMs Facilitate Enamel Remineralization by Increasing Ions Concentration and Mimicking Amelogenin

**Figure 3:**
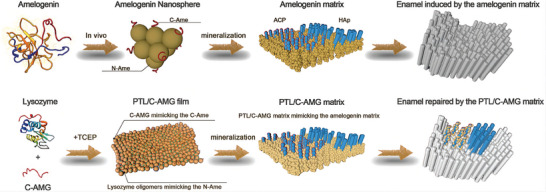
Schematic demonstration of amelogenin and the PTL/C‐AMG matrix (mimicking the N‐Ame and C‐Ame) to mediate the transition from ACP to HAp on enamel for in situ remineralization. Reproduced with permission.^[^ ^33^ ^]^ Copyright 2020, John Wiley and Sons.

The mineralization of enamel entails nucleation and growth of mineral crystals (i.e., HA), which are formed by calcium and phosphorus ions under the initiation of nanoscale amelogenin templates. Thanks to the large specific surface area, high adsorption capability, and excellent designability, NMs can create a microenvironment with high concentrations of calcium and phosphorus ions by degrading or delivering and mimicking amelogenin templates for enamel remineralization acceleration. Therefore, creating a microenvironment with high concentrations of calcium and phosphorus ions and providing or mimicking amelogenin templates can accelerate enamel remineralization. First, since the solubilities of calcium and phosphorus ions in the oral cavity are extremely low, conventional calcium/phosphorus complexes merely could release and offer an effective concentration of effective ions for a limited time to promote mineralization. However, remineralization is a time‐consuming process which requires to be maintained. Only long‐term and continuous administration could achieve efficient remineralization of the target site and eventual successful repair.^[^ ^35^ ^]^ Using nanoscale calcium and phosphorus or loading them into NM carriers (e.g., mesoporous nanomaterials, polymer nanocarriers, and liposomes) can enable a sustained or controlled release, which facilitates the creation of a microenvironment of continual high concentrations of calcium and phosphorus ions.^[^ ^36^ ^]^ For example, the release rates of calcium and phosphorus ions from nanoscale ACP in neutral and acidic solutions are 2–4 times those from non‐nanostructures, and the deposition rates onto the surface of demineralized enamel are also faster.^[^ ^37^ ^]^ In addition to dissolving and releasing calcium and phosphorus ions, nano‐hydroxyapatite (nHA) and its derivatives/analogs (e.g., fluorapatite, zincified apatite, and magnesified apatite) can also bond directly to HA in dental hard tissue, especially at defects such as microcracks and thereby achieve reconstruction efficiently.^[^ ^37^ ^]^ In terms of biomimetic amelogenin, mimicking amelogenin to enhance template function can accelerate the nucleation of HA. However, most previous studies have focused on the cost, limitations, and immunogenicity of allogenic amelogenin, which confound its large‐scale use for remineralization of the enamel.^[^ ^38^, ^39^ ^]^ Recently, NMs produced on a large scale have been used to mimic amelogenin as a template for organic mineralization and to guide the initial nucleation and orientation of crystals effectively.^[^ ^40^ ^]^ For example, phase‐transited lysozymes have a β‐sheet structure similar to that of the amelogenin N‐terminal. The self‐assembled 2D nanointerface can mimic amelogenin to regulate the distribution of nucleation sites, induce ACP to orient and transform into a linear chain structure on the surface, and finally fuse to form acicular and bundle crystals highly similar to natural enamel, which exhibits robust hardness and abrasion resistance (**Figure** **3**).^[^ ^33^ ^]^ In conclusion, NMs may initiate and accelerate enamel remineralization and resolve the challenge of enamel regeneration and slow restoration by both providing mineralization materials and mediating the initiation and growth of enamel crystals.

### 2.2 NMs Facilitate Dentin Remineralization by Preventing Collagen Degradation and Serving as Non‐Collagen Proteins

**Figure 4:**
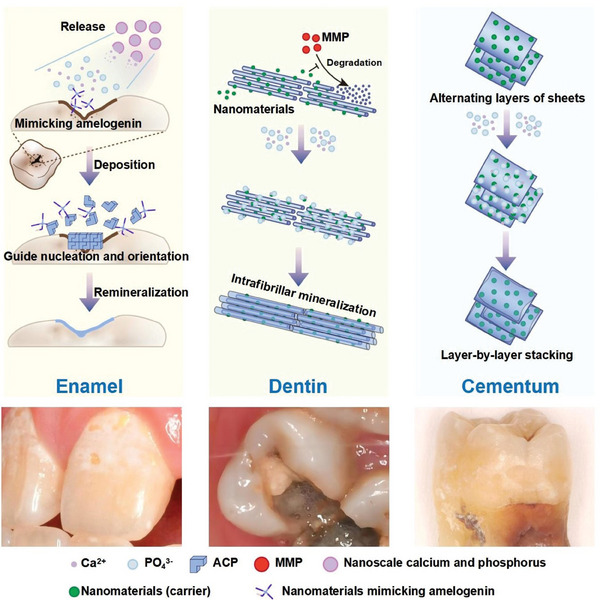
NMs facilitate the mineralization of enamel, dentin, and cementum from a microscopic perspective. a) Nanoscale calcium and phosphorus can enable a sustained or controlled release to create a microenvironment of high concentrations of calcium and phosphorus ions. Other NMs mimick amelogenin to enhance the function of template and guide the initial nucleation and orientation of crystals. b) NMs act as targeted carriers to simulate non‐collagen proteins, transport calcium onto the demineralized dentin collagen, and accelerate remineralization. Protective NMs bind or block the sensitive cleavage sites of collagen to inhibit MMPs and stabilize the stereoscopic structure of collagen fibrils to promote the deposition of ACP at intra‐ and extrafibrillar sites. c) NMs induce the stable adhesion of hydroxyapatite and restore in a layer‐by‐layer stacking manner. MMP: matrix metalloproteinase.

Dentin is mineralized to a lesser degree than enamel and exhibits a higher (20%) content of organic molecules such as collagen.^[^ ^33^, ^41^ ^]^ Unlike enamel, the remineralization of dentin does not depend on the presence of crystal seed.^[^ ^42^ ^]^ Instead, preformed collagen fibers function as adsorption sites and templates for calcium and phosphorus ions to crystalize, resulting in intra‐, inter‐, and extrafibrillar accumulation and mineralization (**Figure** **4**).^[^ ^43^, ^44^ ^]^ NMs not merely maintain the stability of the stereoscopic structure of collagen fibrils by inhibiting the matrix metalloproteinases (MMPs) from degrading them, but also target ACP deposition at intra‐ and extrafibrillar sites by simulating non‐collagen proteins.^[^ ^45^, ^46^ ^]^ MMP inhibitors (such as chlorhexidine and ethylenediaminetetraacetic acid) and chemical crosslinkers used in clinical practice have raised concerns regarding safety and chronic sequelae.^[^ ^47^ ^]^ Protective NMs such as nano‐zinc, nano‐silver, and tiopronin‐protected gold nanoparticles have been used recently to bind or block the sensitive cleavage sites of collagen to inhibit MMP, promote collagen crosslinking, enhance crosslinking density, and prevent collagen microfiber disarrangement. NMs may represent a novel approach to improve the biodegradation resistance of collagen and enhance the mechanical properties and structural stability of remineralized dentin.^[^ ^48^, ^49^ ^]^

Intra‐fibrillar mineralization is the core step of dentin mineralization.^[^ ^33^ ^]^ Steric hindrance and electrostatic repulsion inhibit the localization of calcium and phosphorus ions within collagen fibers and thereby impede regeneration and restoration.^[^ ^50^ ^]^ In natural dentin, the carboxyl groups of non‐collagen proteins (e.g., dentin phosphoprotein and matrix protein) can chelate calcium ions to stabilize and transport amorphous mineralized precursors to intrafibrillar sites and play a role in regulating intrafibrillar mineralization.^[^ ^51^ ^]^ Recombined non‐collagen proteins have been reconstituted or partially extracted to promote biomimetic intrafibrillar mineralization; however, their use has been constrained by limited sources, high cost, and rapid degradation.^[^ ^52^ ^]^ NMs, which can chelate calcium ions and bind collagen, act as targeted carriers to simulate non‐collagen proteins, transport calcium onto the demineralized dentin collagen, and accelerate remineralization.^[^ ^53^, ^54^ ^]^ For example, the ─OH, ─COOH, ─NH_2_ groups of polyelectrolyte NMs represented by PAMAM can absorb calcium and phosphorus ions, while positively charged ─NH_2_ could be targeted to bind to the exposed ─COOH groups of demineralized collagen. It is worth mentioning that ─NH_2_ and ─COOH exhibit higher efficiency in remineralization due to their ability to grab calcium and phosphorus ions than ─OH. Additionally, researchers indicated that positively charged ─NH_2_ may targeted to bind to the exposed ─COOH groups of demineralized collagen, thereby initializing and accelerating the remineralization process. In addition to their deposition on the surface, minerals penetrate deeply and are deposited at intra‐ and interfibrillar sites, resulting in the mineralization of the entire layer of collagen fibers in dentin.^[^ ^25^ ^]^

### 2.3 NMs Facilitate Cementum Remineralization via Structural Mimicking

Cementum is a unique laminated structure composed of alternating layers of sheets of several micron thicknesses that are arranged parallel to the surface of tooth root.^[^ ^55^, ^56^ ^]^ Of the three dental hard tissues, cementum has the highest content of organic compounds. Its degree of mineralization is ≈50%, primarily around collagen. Its chemical composition is characterized by a higher fluorine content than other mineralized tissues.^[^ ^57^, ^58^ ^]^ Fluorine‐containing mineralized collagen fibers are highly aligned in single‐layer sheets that are tightly stacked at a certain angle. The difficulty of mineralization and regeneration of cementum lies in the orderly arrangement of new HA for cementum formation and adherence to the exposed dentin. Due to their excellent designability, plasticity, and stability, NMs have become a potential option for cementum biomineralization since they help repair a highly semblable layered structure and mechanical strength. NMs that mimic the arrangement of alternating layered collagen fibers can construct biomimetic cementum; the silane groups in the flaky silanized nHA fiber can react with multiple hydroxyl groups on the surface of bare dentine to induce the stable adhesion of HA. The structural characteristics of cementum were perfectly restored in a layer‐by‐layer stacking manner; thus the hardness and elastic modulus of the newly formed cementum were 65.43% and 90.7% respectively, higher than those of unstructured HA.^[^ ^59^ ^]^ In addition, the main component of cementum is cellular, and the growth of acellular cementum is a process of gradual mineralization of Sharpey fibers, which are thicker than the dentin collagen fibrils. The mineralization process is like that of dentin; specific mechanisms and principles are described below.

### 2.4 NMs Promote Remineralization through Antibacterial Effects

In the bacteria‐rich oral cavity, biofilm adhering to the teeth surface continuously produces acid, resulting in perpetual mineral loss so that the remineralization process is slowed down or even ceased. Owing to their large surface area, excellent loading capacity, designability, and specific optoelectronic performance, NMs exhibit excellent antibacterial efficiency and targeted and intelligent antibacterial effects. The combination of remineralizing NMs and antibacterial NMs benefits constant and stable remineralization. The antibacterial NMs in remineralization can be concluded by several aspects. First, NMs increase the remineralization quantity through enhanced antibacterial efficiency. Only half a dosage of BG was required to form equal amounts of new mineralized crystals after combining with nano‐chitosan.^[^ ^60^ ^]^ Second, NMs can serve as drug delivery systems to continuously release loaded antibacterial and remineralizing drugs, thus simultaneously preventing demineralization and promoting mineral deposition. For example, scholars designed an epigallocatechin‐3‐gallate and poly(allylamine)‐stabilized amorphous calcium phosphate codelivery hollow mesoporous silica nanosystem (E/PA@HMS) and revealed that biofilm formation was repressed and dentinal tubules were then effectively occluded. Third, with the development of nanotechnology, the controllable antibacterial effects of NMs are realized by endogenous stimuli‐responsive (e.g., masticatory force and pH) and exogenous stimuli (e.g., photodynamic, electrical, and magnetic), among which pH‐stimulated NMs were widely studied. For example, the synthesis of 3‐maleimidopropionic acid‐poly(ethylene glycol)‐block‐poly(_L_lysine)/phyenylboronic acid (MAL‐PEG‐b‐PLL/PBA), tannic acid (TA), and sodium fluoride (NaF) would shape a nanoscale core‐shell structure (PMs@NaF) and undergo acid cleavage since boric acid ester bond formed by the MAL‐PEG‐b‐PLL/PBA and TA was pH‐sensitive, thus allowing the inner NaF to release for remineralization.^[^ ^3^ ^]^

Moreover, intelligent and controllable antibacterial effects can also be achieved by exogenous stimuli. For example, a study has designed chitosan (QCS)‐coated nHAP loaded with chlorin e6 (Ce6), named Ce6@QCS/nHAP, to explore its photodynamic activity. It turned out that 30 min of light irradiation eradicated the dental plaque eradication and inhibited the weight loss of tooth minerals.^[^ ^61^ ^]^ Besides light, the magnetic field is also been applied for cariogenic biofilm ablation. Some scholars have added acrylate‐functionalized iron nanoparticles (AINPs) and iron oxide nanoparticles (IONPs) with antibacterial dimethylaminohexadecyl methacrylate (DMAHDM) and remineralizing ACP nanoparticles.^[^ ^62^ ^]^ Applying a 3 min magnetic force can improve the infiltration of the DMAHDM AND ACP, thus allowing biofilm and pH control, as well as high levels of ions release. It is undeniable that NMs have great potential in dual‐functional (including antibacterial and remineralization) drug research since they exhibit distinct advantages in high electrical conductivity, reaction rate, and distinguished mechanical and chemical properties due to their small size effect, quantum size effect, and surface and interface effects.

## 3 Mechanisms of NMs in Optimizing Biomimetic Mineralization

### 3.1 Providing Calcium and Phosphorus Ion Reservoirs to Maintain Local Supersaturation is the Basis of Biomimetic Mineralization

**Figure 5:**
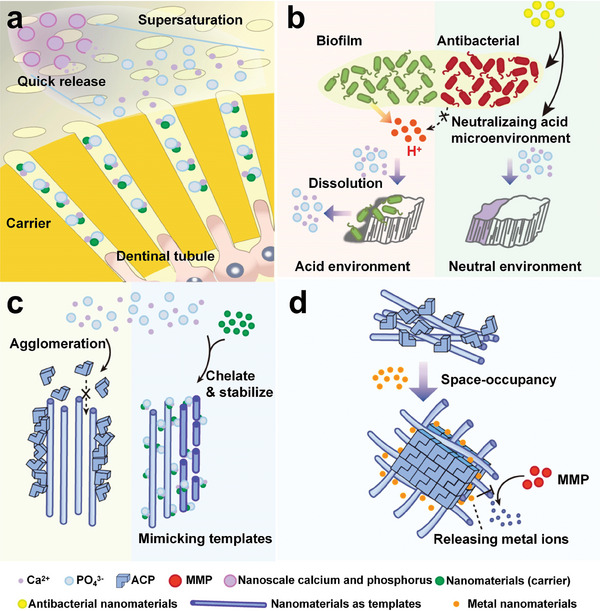
Mechanisms of NMs in optimizing biomimetic mineralization. a) Providing calcium and phosphorus ion reservoirs to maintain local supersaturation is the basis of biomimetic mineralization. NMs remineralization drug delivery systems achieve long‐term controlled release of calcium and phosphorus ions and prevent the early sedimentation onto the surface layer. b) Neutralizing acid microenvironments and improving solubility resistance to inhibit demineralization and the dissolution of newly deposited hydroxyapatite crystals. c) Mimicking or assisting nucleation templates to stabilize ACP and accelerate nucleation and orderly deposition. d) Stabilizing the 3D structure of collagen and enhancing the regulation of crystallization and topology. NMs can maintain the spatial arrangement of liquid collagen or act as competitive inhibitors or inactivators of MMPs to protect collagen.

Sufficient calcium and phosphorus ion concentrations are necessary for the initiation of remineralization; long‐term ion replenishment and the maintenance of supersaturation can facilitate the penetration of calcium and phosphorus ions into deep dental hard tissue (**Figure** **5**a).^[^ ^63^ ^]^ Applications of calcium and phosphorus nanocompounds and nano‐carriers enable the sustained, controlled, and cyclic release of calcium and phosphorus.^[^ ^64^ ^]^ First, homologous calcium and phosphorus nanoscale compounds are released more completely and faster per unit of time; the release of calcium ions from calcium phosphate nanoparticles is approximately seven‐fold faster than that afforded by micron‐sized complexes.^[^ ^65^, ^66^ ^]^ Simultaneous treatment of enamel with nano‐HA and micro‐HA solution also reveals that the former reconstructs enamel thickness twice as much as the latter.^[^ ^67^ ^]^ Second, NM remineralization drug delivery systems such as mesoporous NMs (e.g., mesoporous silica and mesoporous calcium silicate) and nano‐polymers (e.g., PAMAM and poly(carboxybetaine acrylamide) [PCBAA]) readily achieve long‐term controlled release of calcium and phosphorus ions and enhance mineralization. Mesoporous silica nanoparticles (MSNs) have strong acid resistance and high specific surface areas and are the most widely studied nanocarriers in the field of remineralization. They regulate the release and action time of calcium and phosphorus by adjusting their size and adapting the diameters of their surface mesoporous pores, thereby preventing early sedimentation onto the surface layer.^[^ ^68^, ^69^, ^70^ ^]^ The mineralization effect of bioactive glass (BG) applied twice daily for seven consecutive days can be achieved by a single administration of MSN‐loaded BG.^[^ ^71^, ^72^ ^]^ In terms of slow and controlled release, simple Ca^2+^/PO_4_^3−^ is easily adsorbed on the remaining HA template and tends to deposit and mineralize at the tubule orifice in advance. However, Ca^2+^/PO_4_^3−^@MSNs can penetrate into the deeper dentin tubules and release Ca^2+^/PO_4_^3−^, which doubles the depth of mineralization to 25 µm and obtains a better sealing of the dentinal tubule.^[^ ^73^, ^74^ ^]^ With the further addition of pH‐sensitive polyethylene glycol (PEG) as a response medium, the release rate of Ca^2+^/CO_3_^2−^@PEG‐MSNs can double under acid cariogenic compared to neutral conditions, achieving the rapid release of calcium and phosphorus ions for both acid neutralization and deposit to stopping demineralizing and repairing tooth, respectively.^[^ ^75^ ^]^ Though mesoporous silica has been widely studied due to its easy preparation, stable properties, and good biocompatibility, it needs further modification by organic functional groups to perform desired drug‐loading functions.^[^ ^18^, ^71^ ^]^ However, mesoporous calcium silicate uses the surface calcium ions to bind to the loaded drugs (calcium phosphate, antibacterial polyphenols, etc.) through chelation, hydrogen bonding, and electrostatic action, thus achieving higher drug‐loading capacity and long‐term remineralization performance.^[^ ^76^, ^77^ ^]^ In addition, calcium silicate exhibits better antibacterial properties than silica, which may be associated with the ability to alkalize the surrounding environment.^[^ ^78^ ^]^ Thus, calcium silicate should be proposed as the preferred material when constructing multifunctional mineralized drug‐loaded nanosystems. In addition, polymer NMs containing internal cavities [e.g., poly (lactide‐co‐glycolide) (PLGA), PAMAM] are also more commonly used nanoplatforms for the loading and slow release of calcium and phosphorus.^[^ ^79^ ^]^ Nano‐PLGA can enter dentin tubules directly and release calcium and phosphorus ions slowly with the gradual degradation of PLGA, which can sustain ion release for more than one month and promote the mineralization of deep tubules.^[^ ^80^ ^]^ Studies of calcium and phosphorus nano‐carriers currently focus on the above two NMs and their derivatives, while research on other types of materials is still limited. For example, metal‐organic frameworks (MOF) and tubular nano‐carriers (e.g., halloysite nanotubes) also exhibit good loading function;^[^ ^81^ ^]^ however, the application of these carriers in the field of remineralization is still in its infancy, possibly because of the need to certify the in vivo safety of metal ions and organic ligands. Another hindrance may be the excessively rapid ion release of tubular nanocarriers. Consequently, the stability and sustained release capacity of the carrier must be further improved.

### 3.2 Neutralizing Acid Microenvironments and Improving Solubility Resistance to Impede Demineralization and Promote Remineralization

NMs that inhibit the proliferation of cariogenic bacteria and/or enhance the hardness of dental hard tissue can decelerate demineralization and promote remineralization by neutralizing the acidic microenvironment and increasing the resistance of hard tissue to H^+^ (Figure 5b). Cariogenic bacteria are constituents of the commensal microflora. The reduction of local cariogenic biofilms can inhibit demineralization and the dissolution of newly deposited HA crystals, thereby facilitating the mineralization equilibrium reaction and enhancing mineralization efficiency and the hardness of remineralized tissue.^[^ ^82^, ^83^, ^84^ ^]^ Traditional antibacterial drugs often raise concerns regarding microbial resistance and side effects. The antibacterial mechanisms of NMs include membrane disruption, oxidative stress, and DNA damage, thereby exerting excellent antibacterial effects while conferring a low risk of drug resistance.^[^ ^85^, ^86^, ^87^ ^]^

NMs containing metals and their oxides NM (e.g., Ag^86^, Zn^88^, and nTiO_2_^89^) and natural organic antibacterial NMs comprise antimicrobial drugs that are widely used to promote remineralization. Nano‐argentum (nAg) has a broad antibacterial spectrum and good safety. Its association with calcium‐phosphorus complexes can reduce the dissolution of calcium‐phosphorus through its antibacterial action. A dentifrice containing nAg increased in vitro absolute mineral deposition in deciduous enamel by over 10% compared to the control preparation.^[^ ^90^ ^]^ A low concentration (<40 µg mL^−1^) of ZnO NMs can effectively eradicate *Porphyromonas gingivalis* and *Actinomyces naeslundii* in the root tip of teeth, thereby protecting the mineralization formation of cementum.^[^ ^91^ ^]^ Beneath the cariogenic biofilm, the mineralized layer formed by HA alone can only cover <50% of the original demineralized morphology. nTiO_2_ can exert a photodynamic effect after exposure to a light‐emitting diode and thereby remove cariogenic biofilm rapidly. In an in vitro study of a nTiO_2_‐HA composite, the formed mineral layer almost completely covered the enamel surface, while the Ca/P molar ratio increased by 44% compared with the treatment of HA alone.^[^ ^89^ ^]^ Several natural organic molecular NMs also exhibit antibacterial properties and excellent safety. For example, chitosan can form stable covalent bonds with dentin and inhibit the proliferation of *Streptococcus mutans*, thereby improving the stability of new HA in hard tissue. Chitosan nanoparticles increased the calcium‐phosphorus ratio of the total mineral content to 1.85 and the microhardness by 50%.^[^ ^92^, ^93^ ^]^ Merely 2.5 mm FACP NMs are sufficient to fill the collagen lamellae and obtain biomimetic cementum with enhanced acid stability, which is attributed to the structural strengthening of fluorinated biomineralization.^[^ ^94^ ^]^ In addition, rigid NMs with small particle size and strong acid resistance (e.g., strontium and gallium nanoparticles) can also provide interfacial bonding with demineralized structures, and strengthen acid resistance and mechanical properties of newly assembled remineralized HA.^[^ ^95^, ^96^ ^]^ The incorporation of nano‐strontium into nHA has enhanced the crystallinity of nHA and reduced its particle size, subsequently increasing the hardness of remineralized enamel.^[^ ^97^ ^]^ Nano‐strontium‐doped HA at 10^−4^, 10^−3^, and 10^−2^ m concentrations significantly improved the hardness of newly formed enamel in an acidic solution with a pH above 3.5. The degree of improved hardness was directly related to nano‐strontium concentration.^[^ ^98^ ^]^

**Figure 6:**
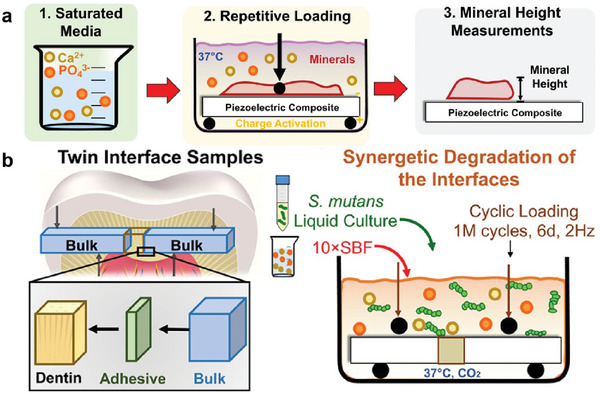
a) Mineralization effects of piezoelectric resin composites. Schematics of an in vitro model to grow and measure minerals on samples subjected simultaneously to supersaturated solutions of simulated body fluid and repetitive mechanical loading. b) Synergistic model to evaluate the bonding strength of dental composites. Schematic diagram showing the preparation of twin interface samples and material testing for bonding strength. Reproduced with permission.^[^ ^99^ ^]^ Copyright 2021, American Chemical Society.

Novel materials such as responsive piezoelectric NMs and nanozymes have been recently developed to provide controlled antibacterial activity and prolonged mineralization. Piezoelectric nanoparticles of barium titanate (BaTiO_3_) exhibited antibacterial properties through the generation of electrical polarization induced by cyclic masticatory forces. Under force stimulation for seven days, piezoelectric BaTiO_3_ nanoparticles eliminated 99% of planktonic bacteria, inhibited bacterial adhesion by 70%, and reduced biofilm formation by 90%. The prevention of bacterial penetration into the remineralized layer led to a 4‐fold increase in mineralized layer surface area compared to that of the non‐piezoelectric control composite (**Figure** **6**).^[^ ^99^ ^]^ Nanozymes such as Fe_3_O_4_ and CoPt catalyze the production of highly reactive oxygen species (ROS) with a high threshold. ROS effectively eliminates cariogenic biofilms that adhere to the enamel surface and improves the deposition efficiency of calcium and phosphorus, thereby reducing HA demineralization.^[^ ^100^, ^101^ ^]^ Nanozymes can inhibit dental cariogenic biofilm, reduce demineralization, and promote mineralization; however, studies of their direct effect on remineralization and the repair of dental hard tissue are still in the initial stages. In the future, mineralization may be greatly accelerated by the enhancement of the trigger and control sensitivity of piezoelectric material and by the efficient catalytic activity of nanozymes.

### 3.3 Mimicking or Assisting Nucleation Templates to Stabilize ACP and Accelerate Nucleation and Orderly Deposition

Although the sufficient bioavailability of calcium and phosphorus is essential for the mineralization of dental hard tissue, the presence of the organic template/scaffold is the primary initiator of mineralization. Mineralized template constituent units, such as amelogenin and collagen fibers, have plate‐like or columnar shapes and a fluffy and ordered 3D structure with self‐assembly properties. Their induction of mineral crystal orientation and arrangement is of paramount importance in determining the structural integrity and functional properties of remineralized tissues (Figure 5c). On the one hand, NMs with larger specific surface areas and smaller particle sizes can chelate and stabilize ACP, which is prone to agglomerate and deposit irregularly during nucleation around the template and form inadequate mechanical properties, thus NMs enable the deep penetration of ACP into the hard tissue for effective remineralization. At the same mass, micron‐sized carboxymethyl chitosan (CMC) can only chelate 5% of ACP by molecular weight, while CMC/ACP nanocomposites with a particle size of only 50 nm can chelate more than 20% of ACP. This leads to three‐fold and five‐fold increases in remineralized enamel and dentin thickness, respectively, compared to that achieved with CMC alone.^[^ ^14^, ^52^, ^102^ ^]^ Doping nMgO in ACP stabilizes ACP and improves the affinity and wettability of ACP for collagen by taking advantage of the stronger hydration of Mg^2+^ compared to Ca^2+^. This, in turn, promotes intrafibrillar lattice mineralization instead of mere surface accumulation of calcium‐phosphorus crystals.^[^ ^103^, ^104^ ^]^ In addition, when the quantity of ACP is constant, the more phosphorylation sites on the surface, the faster they will be bound to the mineralized template of collagen fiber, resulting in more efficient nucleation and deposition.^[^ ^5^ ^]^ The application of phosphate‐rich NMs can introduce additional phosphate functional groups, which stabilize calcium ions and facilitate their transport to the hydroxyl groups of collagen for bonding. For example, pretreatment with sodium trimetaphosphate NMs may increase the contact area between collagen and ACP, thus enhancing and accelerating the binding of calcium phosphate to collagen and finally achieving remineralization in half the time.^[^ ^105^ ^]^ Synthesize peptide/hydroxyapatite nanocomposites have CEMP1 with glycine, glutamic acid, and glutamine, which could bind Ca^2+^ selectively and promote the growth and specific orientation of HA, resulting in stable mineral crystals on the cementum surface.^[^ ^39^, ^106^ ^]^ Black phosphorus nanosheets, composed solely of elemental phosphorus, exhibit a similar effect by degrading to create a phosphate‐rich microenvironment that retains and stabilizes Ca^2+^, and also by adsorbing onto the surface of demineralized collagen fibrils to enhance the stability of the remineralized layer and increase surface potential energy to thereby accelerate remineralization.^[^ ^107^ ^]^

On the other hand, NMs with properties like tunable surface charge and hydrophilicity can mimic the template or enhance its function to promote reactions of calcium and phosphorus with the template and accelerate the nucleation of mineralization. 2D NM graphene oxide (GO) exhibits a planar shape similar to the mineralization template. Its active carboxylic acid and hydroxyl groups chelate Ca^2+^ to form GO‐ACP complexes and guide the orderly deposition and growth of ACP.^[^ ^108^, ^109^ ^]^ GO‐HA and alginate polyelectrolyte complexes act as primers of calcium and phosphorus ions and as templates to induce crystal deposition and fusion into a bundled prismoid‐structured enamel. Compared to the physical stacking of crystal blocks triggered by HA alone, this combination improves the hardness of nascent enamel by ≈30%.^[^ ^110^ ^]^ Furthermore, the mechanical properties of regenerated enamel are closely associated with the precise orientation of crystal growth. ACP and crystal may be reconstructed along the *c*‐axis by materials that mimic the hydrophilic C‐terminal of amelogenin. This orientation control mechanism effectively limits the plastic deformation of individual units within the enamel when subjected to external forces, and enhances the strength of remineralized tissue.^[^ ^28^ ^]^ Current research in this area focuses on bionic polypeptides and has been summarized in a previous review.^[^ ^15^ ^]^ However, recent studies have revealed that NMs can exhibit similar effects. For example, the β‐sheet structure of a phase‐transited lysozyme can simulate the N‐terminal amelogenin peptide (N‐AME) with a central structural domain. Phase‐transited lysozymes can serve as templates to guide the transition of ACP into parallel aligned acicular structure crystals along the *c*‐axis, exhibiting a hardness (4.17 ± 0.32 GPa) and elastic modulus (83.6 ± 4.0 GPa) closer to those of natural enamel than those obtained by fluoride application.^[^ ^33^ ^]^

### 3.4 Stabilizing the 3D Structure of Collagen and Enhancing the Regulation of Crystallization and Topology

The 3D structure of collagen fibers in dentin and cementum serves as the fundamental framework for the development of its unique elastic modulus. ACP undergoes mineralization along the pre‐established axis of liquid collagen, resulting in the formation of remineralized tissue that possesses load‐bearing capacity (Figure 5d). On the one hand, collagen without a well‐organized 3D structure can only promote the haphazard deposition of calcium and phosphorus crystals on its surface, leading to inadequate restoration of the elastic modulus and compromising the ability of dentin and cementum to buffer external pressure. First, the utilization of small particle‐sized degradable NMs (e.g., liposomes) can maintain the spatial arrangement of liquid collagen through space‐occupancy effects to prevent collapse into a planar structure. Subsequently, the gradual degradation of liposomes creates space for the entry of calcium and phosphorus ions.^[^ ^111^ ^]^ Second, carbon‐based NMs with high rigidity may interact with collagen through the formation of carbon‐carbon bonds. This interaction enhances the mechanical strength of the 3D structure and improves the resistance to the collapse of reconstructed collagen. Carbon nanotubes (CNTs) and collagen are both columnar macromolecules that can combine with each other to form a helical winding structure, preventing the collapse of collagen and improving water solubility. Functionalized groups of CNTs (such as amide and carboxyl groups) attract calcium and phosphorus ions to facilitate their orderly binding onto the collagen surface. As a result, the mechanical strength of the mineralized layer is doubled (40 MPa) compared to the control group within the same time span.^[^ ^112^, ^113^ ^]^ GO also exhibits a similar effect when combined with multi‐walled carbon nanotubes, which adsorb onto the collagen surface, identify carbonate/phosphate groups, and enhance its deposition; furthermore, the increased carboxyl groups simultaneously promote calcium deposition. The higher ratio of carbonate/phosphate minerals than that of the control composite indicates an increased amount of bioavailable minerals.^[^ ^114^, ^115^ ^]^ In addition, hydrophilic and easily modified metal‐based NMs can also facilitate the creation of a hydrophilic environment within collagen, which enables collagen fibrils to be fully hydrated and stretched, leading to the rapid infiltration and mineralization of liquid ACP. Tiopronin‐protected gold nanoparticles can cross‐link with collagen extensively, resulting in a denser and more regular arrangement of demineralized collagen fibrils. This cross‐linking leads to a remarkable three‐fold enhancement of the mechanical strength of collagen compared to that achieved with artificial saliva treatment alone, thus improving the strength and stability of guided recrystallized dentin.^[^ ^48^ ^]^

On the other hand, demineralized collagen fibrils exhibit reduced mechanical properties, are susceptible to degradation by MMPs, and lose their capacity to serve as mineralizing templates. NMs capable of releasing metal ions (e.g., Cu^2+^ and Zn^2+^) after degradation can function as competitive inhibitors or inactivators of MMPs, and may thereby play a role in protecting collagen.^[^ ^116^ ^]^ Copper‐doped BG NM slowly released Cu^2+^ within 28 days, inactivated more than half of MMP through metal chelation, facilitated the deposition of calcium ions (Ca/P ratio increased from 1.28 to 1.63), and thus enhanced the strength of collagen.^[^ ^117^ ^]^ Zn^2+^ and Ag^+^ released from ZnO and Ag NMs can competitively prevent the binding of MMPs to Ca^2+^ in collagen fibers, thereby inhibiting enzymolysis. This indirect mechanism maintains the mechanical strength of collagen fibers and enhances its regulation of crystal deposition.^[^ ^118^, ^119^, ^120^ ^]^ In addition, preserving the regulatory function of collagen on crystal deposition is crucial for the mineralization of the bonding interface and the modification of dentin adhesives. Demineralized dentin collagen fibrils typically lose intermolecular cross‐linking exhibit gaps, and appear as broken fragments with dispersed morphology at the peeling interface, resulting in reduced bonding strength. The incorporation of nanoparticles that inhibit MMP activity may maintain a high width (100–200 nm) as well as the typical periodic pattern of 67 nm. Thus, mineralization and bond strength at the resin‐dentine bonding interface may be enhanced.^[^ ^121^ ^]^ Fluoride‐containing zinc and copper fluoride (ZCF) nanoparticles (approximate diameter 100 nm) incorporated into adhesive competitively chelate binding sites within the catalytic regions of MMP‐2, MMP‐8, and MMP‐9, and also stimulate the secretion of endogenous tissue MMP inhibitors. Furthermore, the release of fluorine from the ZCF nanocomposite further reduces MMP‐20 activity. Inhibition of the MMP family protected collagen at the bonding interface from degradation, enhanced the induction of tissue remineralization, and resulted in the formation of a thicker bonding mixed layer than that afforded by a control composite. Consequently, the strength and durability of the mineralized bonding interface are significantly improved. The mechanisms by which NMs optimize the biomimetic mineralization of dental hard tissues are shown in Figure 5, and these mechanisms are summarized in **Table** **1**.^[^ ^119^, ^122^ ^]^

**Table 1:** Mechanisms and potential applications of NMs in biomimetic mineralization of dental hard tissue.

Ca^2+^ and/or PO_4_^3−^ sources	NMs	Size	Form of NMs	Mechanisms of NM for remineralization	Repair time	Thickness/Hardness	Potential Applications	Reference
Artificial saliva	lyso‐PEG	30 nm	Solution	Regulating the nucleation and growth of mineral crystals by interacting with abundant functional groups and mineral ions	7 d	60 ± 5 µm	Dentin hypersensitivity	[128]
Artificial saliva	PAMAM‐NGV@galardin	16.8 nm	Solution	MMP inhibition; formation of domain areas and restricting collagen movement, favoring collagen crosslinking	20 d	46.07 ± 10.94 µm	Dentin caries	[130]
Artificial saliva	PLGA/GSE	105.8 ± 10.5 to 195.4 ± 23.8 nm	Adhesive	1. Promoting collagen crosslinking 2. Inhibiting collagenase 3. Enhancing biodegradation resistance of dentin collagen matrix	1 week, 1, 3 months	5 µm	Collagen crosslinkers	[123]
Artificial saliva	PTL/C‐AMG	50 nm	Solution	1. Mimicking the natural amelogenin matrix 2. Providing physical support and facilitating the oriented arrangement of ACP and its transformation to ordered enamel‐like HAp crystals	10 d	2.0–2.8 µm/4.17 ± 0.32 GPa	Enamel caries	[33]
Simulated body fluid	BaTiO_3_	200 nm	Resin	1. Antibacterial effect 2. Resistance of cyclic mechanical loading 3. Electrostatic interactions attracting oppositely charged ions onto surfaces to induce mineralization	7 d	5–23 µm	Dental resin	[99]
NM	ACMP	180–440 nm	Gel	Continuous ion release	28 d	2–6 µm	Dentin hypersensitivity	[125]
NM	BGN@MSN	MSN (300–400 nm; 150–250 nm), BGN(30–50 nm)	Slurry	1. Controlling the long‐term release of BGN 2. Resistance to dietary acid	30 d	4 −5 µm	Dentin hypersensitivity	[71]
NM	Ca^2+^/PO_4_^3−^@MSNs	50–80 nm	Slurry	Slow and long‐term ion release	–	105 µm	Dentin hypersensitivity	[74]
NM	CaF_2_	71.71 nm	Adhesive	Reconstructing demineralized dentin matrix into a novel porous structure, occupying the sites of the negatively charged groups of NCPs and depressing the NCP polarity	1 d, 12 months	4.78 ± 0.23 µm/0.19 ± 0.03 GPa	Dentin adhesives	[129]
NM	CMC/LYZ‐ACP	200 nm	Gel	Rapid releasing of ions	7 d	4–8 µm	Dentin hypersensitivity	[127]
NM	CPICs	1.6 ± 0.6 nm	Solution	Producing a precursor layer to induce the epitaxial crystal growth of enamel apatite, mimicking the biomineralization crystalline‐amorphous frontier of hard tissue development	48 h	2.7 µm/3.84 ± 0.20 GPa	Enamel regeneration	[30]
NM	CS‐HA	200 nm	Varnish	1. Antibiofilm activity 2. Inhibiting bacterial adherence	24 h	19.55 ± 0.64 µm	Seal root and dentin	[92]
NM	DMAHDM‐NAg‐NACP	NAg (2.7 nm) NACP(116 nm)	Root canal sealer	1. Releasing Ca^2+^ and PO_4_^3−^ 2. Antibacterial effect 3. Acid neutralization	1 month	0.516 GPa	Root canal sealer	[82]
NM	E/PA@HMS	408.4 nm	Solution	1. Quick release of minerals during the first 7 days 2. Sustained slow release from 7 to 28 days 3. Inhibiting adhesion and biofilm formation of cariogenic bacteria	28 d	13 µm/0.601 ± 0.046 GPa	Dentin caries and hypersensitivity	[124]
NM	GO‐CaP	GO (1–2 nm) CaP (<100 nm)	Adhesive	1. Releasing Ca^2+^and PO_4_^3−^ 2. Antibacterial effect	7 d	–	Dentin adhesive	[108]
NM	GO‐HA	–	Solution	Facilitating crystal assembly into an enamel‐like prismatic structure with a highly organized orientation preferentially along the c‐axis	4 d	81.3 µm	Enamel‐inspired biomaterials	[110]
NM	MBN@GOQD	500 nm	Slurry	1. Reacting strongly with Ca^2+^ 2. Providing favorable sites for HA formation 3. Releasing Ca^2+^and PO_4_^3−^	5, 10, 30 d	–	Dentin hypersensitivity	[109]
NM	MCS	100 nm	Paste	1. Releasing Ca^2+^ and SiO_4_^4−^ 2. Inducing apatite mineralization 3. Antibacterial effect	7 d	–	Seal the apical root canal of a tooth as an injectable paste	[72]
NM	Mg‐ACP	200 nm	Solution	Inducing chemical gradient in nanocrystalline attachment and realignment, connecting nanorods to reinforce enamel‐like arrays	96 h	6 µm/2.90 ± 0.13 GPa	Artificial Enamel	[132]
NM	PAA‐ACP@aMBG	425 nm	Paste	Increased ion release of ions under decreasing Ph	14 d	62.56 ± 4.98 µm	White spot lesions	[126]
NM	PA‐ACP@AF‐eMSN	200 nm	Solution	Releasing PA‐ACP precursors	5 d	–	Nanofillers in resins	[69]
NM	PAMAM + NACP	NACP (116 nm)	Solution	1. Rapidly releasing ions at low pH values 2. Acid neutralization	21 d	30 µm/0.53 ± 0.04 GPa	Dentin caries	[79]
NM	P‐CaPK	256.37 ± 29.81 nm	Solution	1. Releasing Ca^2+^and PO_4_^3−^ 2. Antibacterial effect	27 d	–	Disinfectant for infected root canals	[80]
NM	PMS@NaF‐SAP	300 nm	Solution	1. Adhering to tooth 2. Identifying cariogenic conditions and releasing drugs at acidic pH 3. Antibacterial effect	7 d	–	Dental caries	[3]
NM	TCN‐HNT	90 nm	Adhesive	1. Attracting Ca^2+^ and PO_4_^3−^ 2. Antibacterial effect	14 d	–	Orthodontic adhesives	[81]
NM	TiO_2_–HAP	≈50 nm	Adhesive	1. Attracting ions 2. Forming new nucleation sites	7 d	15 µm	Dentin hypersensitivity	[73]
–	ZCF	100 nm	Adhesive	1. Antibacterial effect 2. Fluorine release	5 d	–	Multifunctional dental materials	[119]
–	ZnO	–	Adhesive	Protecting sensitive cleavage sites of collagen, reducing the collagen degradation;	3 months	> 5 µm	Etch‐and‐rinse adhesive	[131]

Abbreviations: ACMP: amorphous calcium magnesium phosphate particles; ACP@AF‐eMSN: polyacrylic acid‐stabilized amorphous calcium phosphate loaded with amine activated mesoporous silica nanoparticles with expanded pores; AMG: phase‐transited lysozyme mimicking an N‐terminal amelogenin with central domain (N‐Ame) combined with synthetic peptide; BaTiO_3_: barium titanate piezoelectric; CMC/LYZ‐ACP: carboxymethyl chitosan and lysozyme‐encapsulated amorphous calcium phosphate nanogel; CPICs: calcium phosphate ion clusters; CS‐HA: chitosan‐grafted hydroxyapatite precursor nanocomplex; DMAHDM‐NAg‐NACP: sealer with dimethylaminohexadecyl methacrylate, nano‐silver and nano‐calcium phosphate; E/PA@HMS: epigallocatechin‐3‐gallate/mineralization precursors co‐delivery hollow mesoporous nanosystem; GO‐Cap: graphene oxide/calcium phosphate nanofiller; GO‐HA: graphene oxide‐hydroxyapatite; Lyso‐PEG: lysozyme conjugated with polyethylene glycol; MBN@GOQD: graphene oxide quantum dots coated with mesoporous bioactive glass nanoparticles; MCS: mesoporous calcium silicate; NM: nanomaterial; NP: nanoparticle; PAA‐ACP@aMBG: mesoporous bioactive glass loaded with amorphous calcium phosphate; P‐CaPK: quaternary ammonium silane, calcium and phosphorus‐loaded PLGA submicron particles; PAMAM+NACP: poly(amidoamine)+nano‐amorphous calcium phosphate complex; PAMAM‐NGV@galardin: galardin‐loaded poly(amido amine)‐NGV peptides; PLGA/GSE: chlorhexidine‐loaded PLGA; PMS@NaF‐SAP: plaque‐inspired micellar multidrug delivery system; PTL/C‐ PA‐ BGN@MSN: bioactive glass‐coated mesoporous silica nanoparticles; TCN‐HCT: adhesives with triclosan‐loaded halloysite nanotubes; TiO_2_–HAP: mesoporous titanium dioxide composite hydroxyapatite; ZCF: fluoride‐containing zinc oxide and copper oxide.

## 4 Potential Design Strategies and Applications

Novel NMs are being designed to deal with different scenarios that require biomimetic mineralization. Accordingly, potential application scenarios (including enamel regeneration, dentin hypersensitivity, and so on) of NMs on biomimetic mineralization are summarized in Table 1. When implemented into specific agents and products, these NMs can be broadly divided into preventive and therapeutic categories.

### 4.1 Preventive Nano‐Remineralization Agents

**Figure 7:**
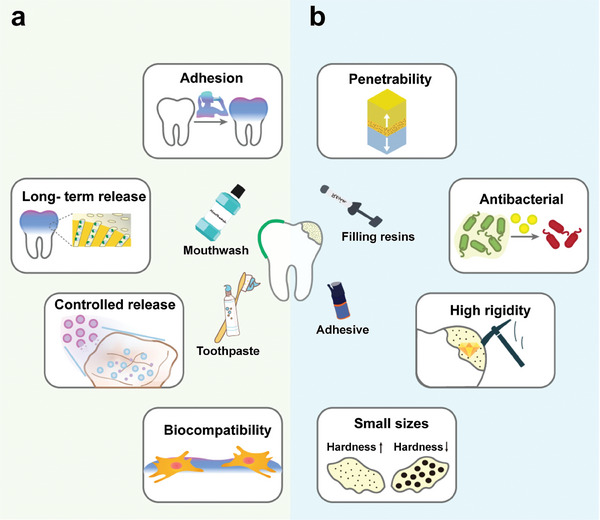
Potential design strategies and applications of NMs in remineralization of dental hard tissue. a) Preventive nano‐remineralization agents. Nanomaterials used in mouthwash, toothpaste, and other oral hygiene products require improved adhesion and stability to ensure long‐term effectiveness. b) Therapeutic nano‐remineralization agents. Nano‐enhanced mineralization materials are utilized primarily as additives to adhesive and filling resins. The permeability, antibacterial property, and mechanical strength of NMs are needed to promote remineralization and mitigate the risk of micro‐leakage between the material and the natural enamel.

Over the past decade, significant advancements have been made in the field of dental hard tissue remineralization.^[^ ^133^, ^134^ ^]^ However, current clinical techniques still face challenges in achieving complete physiologic reconstruction of full‐layer tooth hard tissue. Therefore, from the perspective of long‐term economic benefits, the biomimetic mineralization strategy for prevention remains the primary approach. This strategy focuses on inhibiting demineralization, enhancing HA hardness, and sealing the weak points of hard tissue.^[^ ^135^ ^]^ The application of NMs primarily entails their inclusion into mouthwash, toothpaste, chewing gum, and other prophylactic agents such as pit and fissure sealants and desensitizers (**Figure** **7**a).^[^ ^136^, ^137^, ^138^ ^]^

First, the use of mouthwash, toothpaste, and other oral hygiene products are part of daily routines.^[^ ^139^ ^]^ These products have short dwell times in the oral cavity and require improved adhesion and stability to ensure long‐term effectiveness.^[^ ^140^ ^]^ Currently, studies focus on single or combined applications of fluorine‐containing NMs, nano‐calcium/phosphorus complexes, and antibacterial NMs.^[^ ^141^ ^]^ Through its friction and extrusion effects, toothpaste facilitates the replacement of elements on dental hard tissue surfaces and fills gaps. Toothpaste is particularly suitable for the combined application of NMs with hardness‐strengthening effects (e.g., nano fluorine, metallic nanoparticles) and nHA and its derivatives. Toothpaste containing HA and zinc nanoparticles is more effective in preventing enamel demineralization than fluoride toothpaste alone.^[^ ^137^ ^]^ Sr_0.5_Ca_0.5_CO_3_ nanoparticles possess unique properties by combining the abrasiveness of calcium carbonate with the mineralizing effect of strontium. When subjected to the friction generated by toothbrushing, toothpaste containing this ingredient not only releases strontium to replace calcium and improve the acid resistance of dentin but also provides calcium to promote remineralization.^[^ ^142^ ^]^ The concentration of effective nanoparticles carried by toothpaste is generally highest during brushing and then decreases rapidly. Therefore, focusing on NM affinity to dental surfaces and capacity for sustained release, and on the incorporation of appropriate structural modifications, is crucial during the design process. NMs that bind rapidly to HA within 1–2 minutes of brushing and gargling, that adhere firmly to the enamel surface, and that withstand the buffering effect of saliva are more practical.^[^ ^3^, ^143^ ^]^ Nano‐micelles, constructed by conjugating modified NaF and the highly viscous salivary‐acquired peptide DpSpSEEK, can adhere to enamel specifically and firmly within a few seconds, and withstand more than two days of saliva scour. Based on the pH‐cleavable boronate ester bond, tannic acid is released in response to an acidic microenvironment and subsequently inhibits bacterial adhesion and biofilm formation. Nano‐micelles can also release NaF to promote the deposition of dense mineral crystals, thus constituting a plaque‐inspired micellar multidrug delivery system particularly suitable for incorporation into mouthwash, mouth spray, and other caries prevention products that are susceptible to salivary erosion.^[^ ^3^ ^]^

To address dentine desensitization, nanoscale hollow mesoporous silicon (HMS) was used to load high levels of epigallocatechin‐3‐gallate and poly (allylamine hydrochloride)‐stabilized amorphous calcium phosphate (PAH‐ACP) to build an E/PA@HMS nanosystem with an average particle size of 408.4 nm. It quickly penetrated dentin tubules with diameters <1 µm through gargling and brushing, effectively filling and sealing to a depth of 13 µm, and releasing epigallocatechin‐3‐gallate and ACP over an extended duration (>28 days) to eliminate intratubular bacteria. Because of its sustained release mechanism that promotes the mineralization of dentin collagen, E/PA@HMS may serve as a biomimetic desensitization barrier for exposed dentin.^[^ ^124^ ^]^ Moreover, when considering the more caries‐susceptible preschool and elderly populations, it is crucial to address the potential risk of accidental swallowing. The long‐term safety of preventive nano mineralizers must be ensured; subsequently, the design and utilization of naturally sourced NMs are preferred.^[^ ^124^, ^144^ ^]^ Mouthwash containing a co‐doping of nano‐ACP and naturally sourced chitosan inhibited the adhesion of *S. mutans*, *Streptococcus sanguis*, and *Streptococcus oralis* to the dental surface. Chitosan facilitated the stabilization of ACP with a diameter <40 nm by entering the interior of demineralized defects to enhance mineralization.^[^ ^145^ ^]^ PCBAA/ACP nanocomposites comprised of ACP and zwitterionic PCBAA (50.67 ± 2.37 nm) from dietary sources stabilized ACP under physiologic pH through the interaction of its ─COOH and ─NH_2_ functional groups and inhibited its rapid aggregation and spontaneous transformation. PCBAA also conferred resistance to non‐specific protein and bacterial adhesion, as well as biofilm formation through electrostatic‐induced hydration. In the cariogenic acidic microenvironment, cationization of PCBAA induces bacterial membranolysis and presents ion deposition sites to accelerate internal crystal growth and the formation of dense mineralized layers. Long‐term in vivo experiments have demonstrated the absence of significant side effects.^[^ ^146^ ^]^ Polymer NMs like PCBAA NM and PAMAM NM stabilize ACP by terminal group to chelate calcium and phosphorus ions. Nevertheless, the zwitterionic PCBAA NM does not merely offer ion deposition sites, its specific quaternary amino groups in the polymer chain exhibit highly cationic and bactericidal properties, which is lacking in PAMAM.

**Figure 8:**
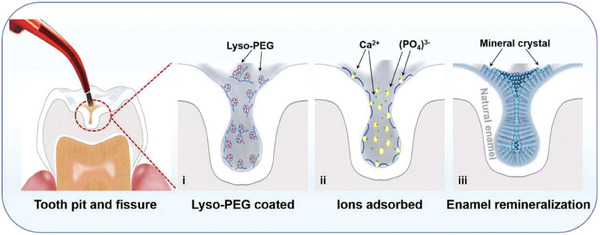
Schematic of the list‐PEG‐induced remineralization process in pits and fissures. The scheme illustrates the rapid formation of lyso‐PEG oligomer nanoparticle‐based nanofilm coatings in the deep zone of pits and fissures, followed by ion adsorption from saliva and spontaneous remineralization toward the formation of enamel‐like HAp structures in pits and fissures to achieve in situ biomimetic sealing. Reproduced with permission.^[^ ^135^ ^]^ Copyright 2022, John Wiley and Sons.

Second, defects such as enamel pits and fissures, perikymata, and dentinal tubules are anatomic structures that facilitate the penetration and diffusion of food residues, microorganisms, and H^+^. More than 50% of demineralization occurs within these areas.^[^ ^147^ ^]^ These narrow and deep structures, along with weakened sites, are susceptible to progressive demineralization. The particle size range of NMs confers a natural advantage in sealing these sites, and those with a size of <30 nm have superior permeation and filling capabilities as pit, fissure, and tubular sealants.^[^ ^71^, ^148^ ^]^ PEG‐phase‐transited lysozymes nanoparticles, featuring a diameter of 23.42 nm, exhibit an impressive penetration depth of 17 000 µm within 30 s, which is more than three‐fold greater than that achieved by a micron‐grade commercial pit and fissure sealant (5000 µm). PEG‐phase‐transited lysozymes can attract calcium and phosphorus ions continuously to aggregate deeply, forming an enamel‐like crystal HA layer with enhanced mechanical stability (**Figure** **8**).^[^ ^135^ ^]^ The utilization of GO quantum dots coated with mesoporous bioactive glass nanoparticles (MBN@GOQD) offers similar advantages. On one hand, hydrophilic functional groups such as ─O and ─OH on the surface of GOQD attract calcium ions avidly, resulting in the immediate formation of calcite clusters in simulated body fluid (SBF) that mainly consists of sodium bicarbonate (NaHCO_3_). With the degradation of bioactive glass coated by GOQD, the acicular‐like HA gradually replaced calcite after 10 days. The dentin tubules of both the crown and root can be completely occluded in the early stage; thus, the mineralization rate is 3–5‐fold higher than that obtained by simple MBN. On the other hand, GOQD exhibits long‐term (>30 days) sustained release of high concentrations of calcium, silicon, and phosphate ions. This prolonged release can also promote the long‐term remineralization of the dentin surface. Consequently, MBN@GOQD emerges as a promising candidate for the treatment of dentin desensitization by providing both physical sealing and durability.^[^ ^109^ ^]^

### 4.2 Therapeutic Nano‐Remineralization Agents

Filling is the primary method for treating parenchymal dental lesions. Bonding and filling techniques play crucial roles as essential components of therapy. Although these procedures have been greatly improved and optimized, persistent challenges include collagen degradation and hydrolysis of resin monomers that may result in the collapse of collagen fibers and the insufficient remineralization of edges. Secondary caries and filling dislodgement resulting from bond failure and fractured filling margins continue to challenge dental scientists and clinicians. Nano‐enhanced mineralization materials are utilized primarily as additives to adhesive and filling resins. Their functions center on reinforcing the strength of the mineralized layer and providing antibacterial activity (Figure 7b).^[^ ^149^, ^150^ ^]^

In terms of adhesives, highly hydrated non‐collagen proteins within the demineralized dentin matrix create an interfacial microenvironment that hinders adhesive penetration into the matrix, consequently compromising long‐term bond durability.^[^ ^151^ ^]^ Therefore, previous bonding failures have been attributed to either the vulnerability of demineralized dentin matrix (mainly collagen fibrils) to hydrolysis, or acid production by residual bacteria at the bonding interface, leading to microleakage.^[^ ^152^ ^]^ In the design of nano‐modified adhesives, a focus on enhancing permeability and inhibiting MMPs is essential to prevent degradation of the collagen matrix, as discussed in section 3.4 above.^[^ ^153^, ^154^, ^155^ ^]^ In addition, the inclusion of antibacterial nanoparticles facilitates remineralization indirectly by reducing bacterial colonization at the bonding interface. For example, orthodontic adhesives supplemented with gallium (Ga)‐doped mesoporous silica nanoparticles at concentrations of 1%, 3%, and 5% released an antibacterial agent (Ga) and mineral ions (Ca^2+^, PO_4_^3−^), resulting in inhibition of *S. mutans* and a dose‐dependent decrease of enamel demineralization of bracket‐bound teeth without compromising adhesive properties.^[^ ^96^ ^]^

Regarding filling resin, the incorporation of hydrophilic nanoparticles with small particle sizes and high rigidity generates a composite resin that exhibits superior mechanical strength compared to conventional resin monomers. This composite may also promote remineralization and mitigate the risk of micro‐leakage between the material and the natural enamel.^[^ ^156^, ^157^ ^]^ However, a consideration of nanoparticle quantities during NM design is essential to maintain a balance among various aspects of function. First, the addition of nanoparticles should not compromise the mechanical properties of the resin. The nanoparticle dosage should be carefully controlled, because excessive doping may impair resin polymerization and reduce durability.^[^ ^158^ ^]^ For instance, in the case of HA/Li‐Biometal organic frameworks (Li‐BioMOFs), doping within the range of 0–60 ppm (wt.%) significantly increased both the flexural modulus and Young's modulus of nanocomposite resin compared to the control resin. At 60 ppm, the values reached 132.5 MPa and 11.5 GPa, respectively. In addition, Li‐BioMOF facilitated the formation of a porous network conducive to adhesion by nHA, prevented its aggregation, and finally led to ordered mineralization. However, Li‐BioMOF concentrations of over 60 ppm resulted in structural collapse due to insufficient polymer support, thus compromising the original mechanical strength.^[^ ^159^ ^]^ Second, the functional balance between mineralization, antibacterial activity, adhesion, and curing properties of resin should be considered comprehensively.^[^ ^160^ ^]^ For instance, the incorporation of triclosan‐loaded halloysite nanotube (TCN‐HNT) filler into resin at varying concentrations (5, 10, and 20 wt.%) conferred dose‐dependent increases of both antibacterial activity against *S. mutans* and mineralization strength. After 72 h, only the resin containing 20%TCN‐HNT inhibited the growth of *S. mutans*. The 20%TCN‐HNT‐supplemented resin also demonstrated the highest degree of mineral precipitation at the adhesive‐enamel interface. However, the inclusion of TCN‐HNT >5 wt.% compromised bonding performance. Therefore, the optimal efficacy of resin nano‐mineralizers relies on balancing their mineralization effect with bonding performance.^[^ ^81^, ^157^ ^]^

**Figure 9:**
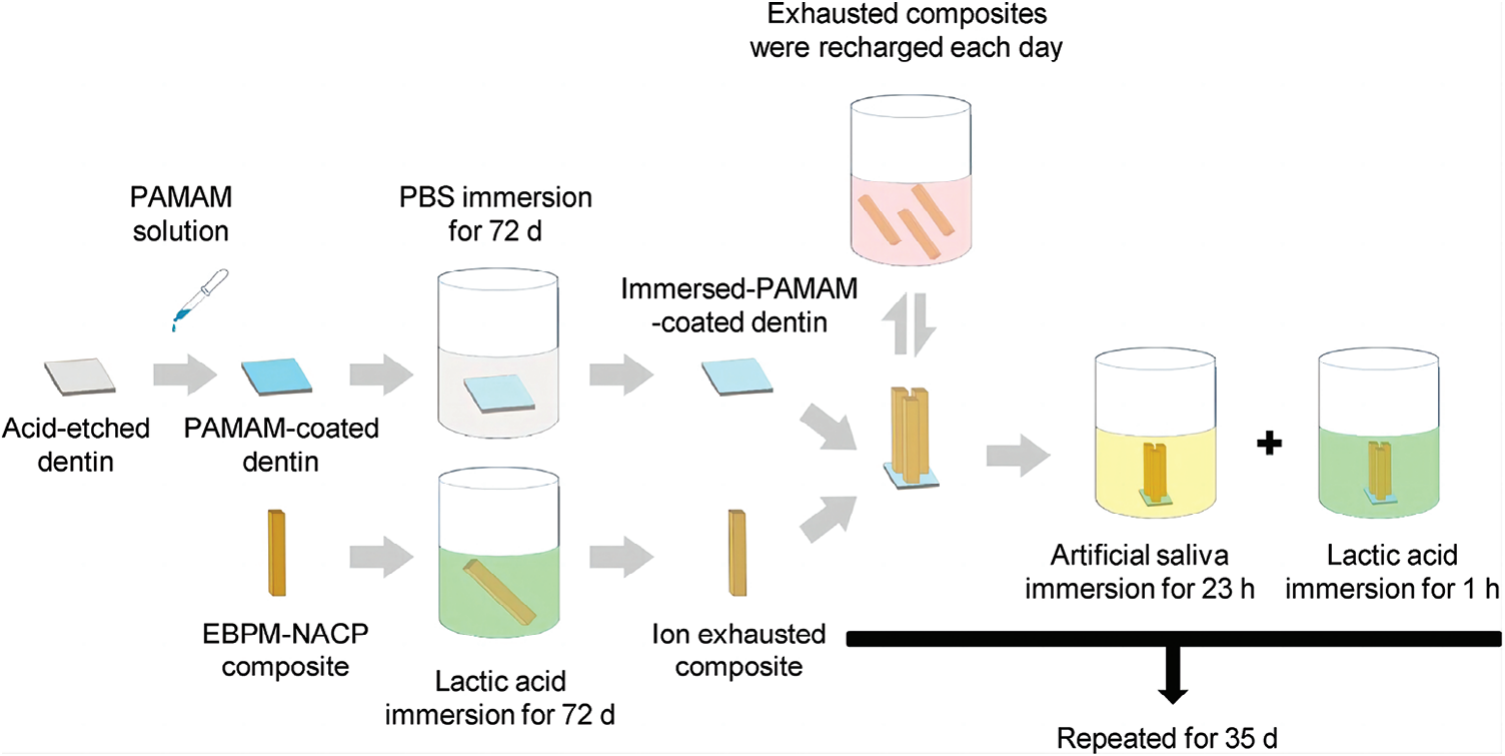
Schematic illustration showing how the PAMAM + EBPM‐NACP composite method was used to induce long‐term dentin remineralization. Reproduced with permission.^[^ ^166^ ^]^ Copyright 2018, Elsevier.

The rechargeable resin PEHB (comprising monomers Pyromellitic glycerol dimethacrylate, ethoxylated bisphenol‐A dimethacrylate, 2‐hydroxyethyl methacrylate, bisphenol‐A glycidyl dimethacrylate) has recently garnered significant attention.^[^ ^161^, ^162^, ^163^ ^]^ These monomers possess abundant carboxylate groups that enable calcium ion chelation, occupy vacant sites within the resin, and release calcium and phosphorus ions by means of concentration differences. In addition, following exposure to a remineralization solution, these ions can be recaptured through electrostatic interaction and space‐occupying effects, thereby exhibiting recyclable charging performance and enhanced remineralization efficiency. The addition of NMs further improves the advantages of this resin. On the one hand, NMs improve the efficiency of release and replenishment. Nano‐ACP‐doped PEHB demonstrated an impressive calcium and phosphorus ion release that was sustained for seven days after only one minute of exposure to the remineralization solution. This remarkable charging efficiency is three‐fold that of the non‐nanoscale calcium and phosphorus complex and is attributed to the small particle size and high surface area of nano‐ACP, which amplify the ion space‐occupying effect of the rechargeable resin.^[^ ^162^ ^]^ On the other hand, incorporating NMs with ionizable groups or the ability to chelate phosphate into rechargeable resins further enhances the recyclable remineralization effect. The amino^[^ ^164^, ^165^ ^]^ groups in PAMAM can be stored in the resin interior by electrostatic interactions between the resin and the remaining dental hard tissue for an extended time span, and can also chelate a significant number of phosphate groups. Immersion and supplementation with calcium and phosphorus components once per week restored the hardness of acid‐etched dentin within 35 days (**Figure** **9**).^[^ ^166^ ^]^ Polymer‐kaolinite (C(K‐acrylamide)) nanocomposite resins contain a substantial amount of ─OH and thus have excellent phosphate adsorption capabilities. Upon incorporation into resin, ─OH was released for up to 2 months. Furthermore, ion release performance was recovered by soaking in a remineralization solution for only 1 min. The application of this nanocomplex with a high discharge–charge ratio significantly increased HA formation on the enamel surface.^[^ ^167^ ^]^ Potential applications of nano‐remineralization formulations are shown in Figure 7.

## 5 Summary and Prospects

In recent years, increasing studies have demonstrated the clinical potential of NMs in dental hard tissue remineralization. For example, in pre‐clinical trials, lysozyme conjugated with polyethylene glycol (lyso‐PEG) NM reduced the depth of demineralization in deep pits and fissures by reducing the amount of plaque and forming newly remineralized layers in 7 participants without causing a remarkable immune response, which reflects the feasible remineralization efficiency of NMs in the real human oral cavity.^[^ ^135^ ^]^ As for in vivo animal models (including rats,^[^ ^146^ ^]^ dogs,^[^ ^168^ ^]^ rabbits,^[^ ^169^ ^]^ pigs,^[^ ^170^ ^]^ etc.), they also reflect the remineralization efficiency of NM well. Take the rats for example, the simply smearing application of nanocomposite (such as PMs@NaF‐SAP) on the rats’ teeth surface would repair demineralized tissue in a highly cariogenic environment by inducing biomimetic remineralization.^[^ ^3^ ^]^ The results of these in vivo studies suggest that NM has sufficient clinical effectiveness in remineralizing. However, most existing in vivo studies couldn't be used as an optimal reference for clinical trials. On the one hand, the mode of action of non‐solid NM is limited to smearing. The difference in the use effect of the same kind of NM in different oral health care products should be verified according to the application scenario. It is difficult to simulate the actual application scenarios of mouthwash and toothpaste in the animal mouth. On the other hand, whether the addition of these NMs to adhesives and resins will affect their mechanical properties such as bonding strength and polymerization degree still needs to be systematically evaluated. The current application of NMs in remineralization is focused on nanocarriers, nano‐ACP, nano‐sized biomimetic proteins and peptides, and the use of dendritic macromolecules as nucleation templates. These advancements pave the way for future research and development, which will continue to prioritize rapid penetration, intelligent controlled release, and long‐lasting slow release.

First, intelligence plays a crucial role in the mineralization process. NMs responding to exogenous stimuli of optics, magnetic field, and masticatory pressure allow their remineralizing ability to be controlled and enhanced. For example, photo‐responsive NM (based on TiO_2_^[^ ^89^ ^]^ and MOF^[^ ^81^ ^]^), magnetic NMs (based on Fe_3_O_4_^[^ ^171^ ^]^), and piezoelectric NM (base on BaTiO_3_^[^ ^99^ ^]^). NMs also recognize endogenous stimuli through surface modification, including recognition of cariogenic acidic environments and oral temperature.^[^ ^155^, ^172^ ^]^ Designing multifunctional NM for different mineralization stages is currently a hot topic in the field of biomimetic mineralization, including but not limited to the identification of tooth surface, antibacterial, template mimicking, ion releasing, and improving solubility resistance. For example, the E/PA@HMS nanosystem exerted an antibacterial effect and remodeled collagen fibers first, and then released calcium and phosphorus ions to promote dentin remineralization.^[^ ^124^ ^]^ Insufficient mineralization occurs at lower ion concentrations, while excessive concentrations lead to unnecessary deposition. Therefore, the exploration of NMs that intelligently deliver the ions required for mineralization is imperative. This remains a significant area for further research. However, accurately determining the “right time” in the clinical setting poses difficult challenges; thus, the development of both smarter drugs capable of self‐monitoring and on‐demand drug delivery is necessary. NMs offer a promising avenue for achieving co‐delivery of multiple components, and can even achieve cascade release of multiple components in distinct environments. This release technology has been widely used in the diagnosis and treatment of various diseases, while not yet being extensively explored in dental remineralization. Cascade drug release may address the diverse requirements of fluoride, biomimetic proteins or peptides, and metalloproteinases in different stages of remineralization.

The second aspect revolves around the multicomponent and multifunctional nature of remineralization. Hard tissue demineralization is a multifactorial disease. Nano‐mineralizing materials with multi‐target design and/or synergistic mechanisms of action hold immense potential for future clinical applications. Such NMs combine antibacterial activity with hardness enhancement or permeability enhancement with MMP inhibition. However, in the oral environment, nano‐mineralizing materials face multiple challenges due to the combined effects of temperature, dental biofilm, salivary proteins, food residues, and bacterial metabolites. These materials must withstand salivary rinsing and the friction caused by mixed food and soft tissue.^[^ ^173^ ^]^ The application of biomimetic principles and methods to prepare high‐performance biomaterials is a practicable strategy for studying how natural creatures live by their structures and functions. One typical example is that mussel‐inspired NMs performed reliable wet adhesion in a dynamic oral environment, thereby effectively transferring calcium and phosphorus to deposit and increasing enamel mineral density.^[^ ^174^ ^]^ Despite advancements, the stimulation of intraoral remineralization is still limited. Therefore, the realization of multifunctionality and the confirmation of the clinical efficacy of nano‐mineralizing materials are essential.

The third aspect focuses on the importance of natural and efficient NMs. The oral cavity is the beginning of the digestive tract. Even though NMs exhibit excellent performance in the field of stomatology in terms of properties, pro‐mineralization capabilities, and designability, a comprehensive synthesis of experimental and clinical data regarding the biocompatibility, toxicity, and biodynamic behavior of nanoscale materials is essential. These obstacles also impede the eventual clinical application of nano‐mineralizing materials. There is a consensus about NMs which are potentially harmful since their small size prompts them to interact directly with biological macromolecules such as proteins, polysaccharides, and DNA.^[^ ^175^ ^]^ The toxicity of NMs is closely related to the amount used. Usually, only small amounts of NMs are applied to the dental hard tissue, and the action time is short, thereby avoiding high risks to cell viability. Moreover, NM remineralizes dental hard tissue mainly by contact rather than directly acting on cells. Since the oral mucosal barriers could protect external substances from internalization, the risks of using remineralizing NMs could be further decreased. From a safety perspective, organic and natural NMs pose less risk to the human body compared to inorganic ones. Surface modification is a commonly used method to improve the biocompatibility, dispersibility, and stability of inorganic NMs. For instance, the citrate coating significantly allowed the controlled release of silver ions from nAg, thus eliminating toxicity effects.^[^ ^77^, ^176^ ^]^ Though the biosafety of remineralizing NM used in dental hard tissue has been verified by various in vitro cell experiments, in vivo animal experiments, and even pre‐clinical trials, it is still necessary to involve biocompatibility evaluation when developing novel NMs. For in vitro cellular experiments, since how NMs interact with cells is complex, the evaluation of oxidative stress and apoptosis in addition to cellular proliferation activity will help to comprehensively estimate potential threats. For in vivo animal experiments, it is encouraged to carry out long‐term toxicity evaluation experiments. Therefore, the development of naturally‐sourced, environmentally friendly, and highly efficient NMs that are effective at low concentrations and dose frequencies should be prioritized. This approach represents a preferred solution to overcome the challenges associated with future clinical implementation.

From traditional non‐calcium phosphate materials to calcium phosphate constructs and from simple classical traditional theory to biomimetic mineralization restorations, scientists have been continuously searching for the optimal approach to achieve the comprehensive restoration of enamel from chemical composition to complex structure. The development and application of NMs in the field of remineralization offer auspicious prospects to expedite this process. Based on the nanoscale structure of teeth, NMs can be designed to restore both the shape and function of dental hard tissue via biomimetic mineralization, whose stability and mechanical properties could be further enhanced during this process.^[^ ^177^ ^]^ From the perspective of material composition, NMs should be programmed to release drugs or ions in a manner that aligns with the regeneration stages of dental hard tissues. From the perspective of material preparation, current techniques require the combination of several general preparation methods to achieve composite properties; thus, how to produce NM in a one‐step, in large quantities, and at low cost remains a crucial issue to be addressed before their widespread adoption. Furthermore, it is essential to explore and identify effective novel NMs. Taking inspiration from the frontier research of bone hard tissue may show a high feasibility.^[^ ^178^ ^]^ In contrast to traditional single‐target treatment strategies such as fluoride and ACP‐based therapies, the future development of remineralizing NMs will encompass the entire remineralization process. In the future, these materials will play diverse roles that include providing essential mineral ions, mimicking the functions of collagen and non‐collagen proteins, inhibiting and activating proteases, and neutralizing bacterially‐induced acidic microenvironments.

## Conflict of Interest

The authors declare no conflict of interest.
